# Inverse Tone Mapping Based upon Retina Response

**DOI:** 10.1155/2014/168564

**Published:** 2014-03-12

**Authors:** Yongqing Huo, Fan Yang, Vincent Brost

**Affiliations:** ^1^School of Communication and Information Engineering, University of Electronic Science and Technology of China, Chengdu 611731, China; ^2^LE2I-CNRS 6306 Laboratory, University of Burgundy, 21078 Dijon, France

## Abstract

The development of high dynamic range (HDR) display arouses the research of inverse tone mapping methods, which expand dynamic range of the low dynamic range (LDR) image to match that of HDR monitor. This paper proposed a novel physiological approach, which could avoid artifacts occurred in most existing algorithms. Inspired by the property of the human visual system (HVS), this dynamic range expansion scheme performs with a low computational complexity and a limited number of parameters and obtains high-quality HDR results. Comparisons with three recent algorithms in the literature also show that the proposed method reveals more important image details and produces less contrast loss and distortion.

## 1. Introduction

Recently, high dynamic range (HDR) image is a considerable topic in image processing fields [1]. The newly developed HDR monitors have greatly extended the limited dynamic range of conventional displays and can simultaneously present bright highlights and dark shadows, so they have gained significant interests in industry. At the same time, the large existing base of low dynamic range (LDR) images makes it necessary to solve how to show LDR images and videos on HDR monitors. This arouses a number of algorithms to expand LDR content to HDR content, which is called inverse tone mapping (ITM) and has been developed as reviewed by Banterle et al. [2]. Among these algorithms, Akyüz et al. [3] indicated that LDR image does not necessarily require sophisticated treatment to produce a compelling HDR experience. Simply boosting the range of an LDR image linearly to fit the HDR display can equal or even surpass the appearance of a true HDR image. This method works well under the hypothesis that the input image is high quality. Masia et al. [4] presented an exponential expansion method with *γ* as exponent, which focuses on images with large saturated regions.

The more sophisticated algorithms detect saturated areas in image and expand them by intricate processing or boost them largely. Meylan et al. [5] proposed a piecewise linear mapping function that allocates more range to those highlights in image. Banterle et al. [6] gave a general framework to map LDR content with saturated areas. The LDR content is first mapped to middle dynamic range by iTMO (inverse tone mapping operator); then, an expand map is computed to reconstruct lost luminance profiles in saturated areas of the image. Rempel et al. [7] performed a similar method; a brightness enhancement map is computed to scale the contrast which has been linearly extended to middle range.

Although the algorithms described above produce appealing results for a wide range of LDR contents, the linear expansion may not work well for images with lower quality. The exponential expansion based on *γ* transmission is unsuitable for images with low key value. Other algorithms perform sophisticated treatment to saturated areas or boost them largely. This introduces the possibility of making the image appear worse than before processing through the introduction of objectionable artifacts, and the large boosting to the bright image areas sometimes results in contouring artifacts for bright object [4].

In fact, because of the large difference between the luminance ranges of these two formats, the faithful reproduction of the HDR content from the LDR content is not possible in general. However, studies on human visual system (HVS) showed that the perceived brightness of each point in a scene is not simply determined by its absolute luminance; instead, the electric signal generated by the retina is transmitted through different layers of cells that introduce a complex, and not yet fully understood, sequence of spatial interactions, nonlinear mappings, and feedback mechanisms [8]. One useful consequence of these mechanisms is that, in order to reproduce an image, it is not necessary to generate an identical or proportional luminance on the display; by exploiting the characteristics of human vision, it is possible to process the image to amplify its dynamic range without producing a significant change in the visual sensation experienced by the observers [8].

In this paper, based on the retina response, a physiological ITM scheme is proposed, which is able to produce high-quality results with a very low computational complexity and a limited number of parameters. The main novel contribution consists in the design of the local adaptive response of retina and its inverse, which comply with the physiological perception procedure to light and minimize the formation of artifacts.

## 2. Proposed Method Presentation

Firstly, the proposed method deduces the local retina response and then inverses it, finally based on the inversed model to expand the dynamic range of LDR images.

### 2.1. Algorithm Description

Compared to the dynamic range of the real-world scene, the response of photoreceptors in retina has a narrow dynamic range, thanks to the adaptation mechanism in human vision, which makes the eyes first adapt to some luminance value and then perceive images in a rather small dynamic range around this luminance value. Thus, the basic process of human vision is a global tone mapping to the entire scene. This global function can be described by the relationship between retina response and stimulus light intensity [9] as

(1) $$\begin{array}{c} \frac{R}{R_{\max}}=\frac{I^{n}}{\left(I^{n}+\sigma^{n}\right)}, \end{array}$$

where *R* (0 < *R* < *R*_max⁡_) is the retina response to the light intensity *I* and *R*_max⁡_ is the maximum response. *σ* is the global adaptation level; it represents the intensity required to generate a response that is one-half the amplitude of *R*_max⁡_. The parameter *n* is a sensitivity control exponent.

It has been proven that the absolute brightness information is of secondary importance to HVS and tends to be largely discarded on very early stages of visual processing through mechanisms of brightness constancy. Local contrasts are used instead to convey the wealth of information about the scene [10]. So, the more proper description of retina response should be a local mapping. The local adaptation property can be realized by changing the global adaptation level *σ* to the local adaptation level *σ*_*p*_ of pixel *p*. Let *I*_*p*_ be the intensity of pixel *p* in real-world scene; the response *R*_*p*_ of the retina is regarded as the intensity of the LDR image perceived by eyes which can be described as

(2) $$\begin{array}{c} R_{p}=\frac{R_{\max}I_{p}^{n}}{\left(I_{p}^{n}+\sigma_{p}^{n}\right)}, \end{array}$$

where *R*_max⁡_ means the maximum value of the LDR output.

For inverse tone mapping, the *I*_*p*_ needs to be solved from (2); the result is as

(3) $$\begin{array}{c} I_{p}={\left(\frac{R_{p}\sigma_{p}^{n}}{\left(R_{\max}-R_{p}\right)}\right)}^{1/n}. \end{array}$$

To avoid zero denominator, a small positive number *δ* is added to (3):

(4) $$\begin{array}{c} I_{p}={\left(\frac{R_{p}\sigma_{p}^{n}}{\left(R_{\max}-R_{p}+\delta \right)}\right)}^{1/n}. \end{array}$$

The *R*_*p*_ and *R*_max⁡_ can be obtained from LDR image directly. The following will describe how to set parameters *n* and *σ*_*p*_.

### 2.2. Parameters Setting

The sensitivity parameter *n* was discussed in the literature [9] that has a value generally between 0.7 (long test flashes) and 1.0 (short test flashes). After carrying out lots of experiments by increasing the value of *n* gradually from 0.7 to 1.0, the results suggest that *n* = 0.9 is better for the test images used in this paper.

The *σ*_*p*_ is local adaptation level; it describes the surrounding intensity information of a pixel. In general, the arithmetic average, the geometric average, or a Gaussian blurred version within a local region of the image can be used for determining this parameter. Here, the local surrounding intensity *I*_*ps*_ of pixel *p* in the HDR image is used to represent the *σ*_*p*_. With only the LDR image, based on the assumption that the maximum luminance 255 of the LDR image is mapped to the maximum luminance of the HDR display, the algorithm first computes *L*_*ps*_ of the LDR image and then multiples it by the ratio between the maximum luminance of HDR display and 255.

There may be various ways for computing *L*_*ps*_. The bilateral filter introduced by Durand and Dorsey [11] is used here. The proposed inverse tone mapping operator is summarized as

(5) $$\begin{array}{c} I_{p}={\left(\frac{R_{p}{\left(I_{ps}\left(\sigma_{m},\sigma_{d}\right)\right)}^{n}}{\left(R_{\max}-R_{p}+\delta \right)}\right)}^{1/n}. \end{array}$$

The output *L*_*ps*_ of the bilateral filter for a pixel *p* is

(6) $$\begin{array}{c} L_{ps}\left(\sigma_{m},\sigma_{d}\right)=\frac{1}{W_{p}\sum_{q\subset \Omega }f_{\sigma_{m}}\left(q-p\right)g_{\sigma_{d}}\left(R_{q}-R_{p}\right)R_{q}}, \end{array}$$

where *W*_*p*_ is a normalization factor:

(7) $$\begin{array}{c} W_{p}=\underset{q\subset \Omega }{\sum }f_{\sigma_{m}}\left(q-p\right)g_{\sigma_{d}}\left(R_{q}-R_{p}\right), \end{array}$$

where *σ*_*m*_ is the standard deviation for a Gaussian *f* in the spatial domain such as

(8) $$\begin{array}{c} f_{\sigma_{m}}\left(p\;|\;p=\left(x,y\right)\right)=K_{m}\exp\left\{-\frac{\left(x^{2}+y^{2}\right)}{\sigma_{m}^{2}}\right\}, \end{array}$$

where  *σ*_*d*_ is the standard deviation for a Gaussian *g* in the luminance domain. *K*_*m*_ is a normalization factor and Ω is the whole image. In the proposed algorithm, *σ*_*m*_ and *σ*_*d*_ are set empirically to 16 and 0.3, respectively.

## 3. Experiments and Evaluations

**Figure 1:**
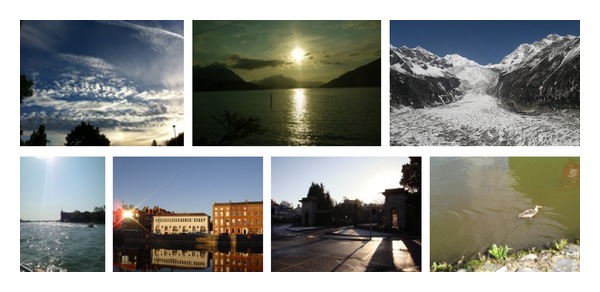
A subset of test images, from top-left to right-down: sky, sun, snow, sea, building, gate, and river.

The proposed algorithm is implemented on a PC (i5-2520, 2.5 GHz). The maximum luminance of the HDR monitor is set to 3000 cd/m^2^, according to the most popular HDR monitor “Brightside's 37.” Figure 1 shows a subset of the test images. These images represent various lighting conditions, from very dark to very bright.

In order to test and validate the performance of the proposed scheme, the other three inverse tone mapping operators are also implemented: Banterle et al.'s inverse photographic mapping (iPG) [6], LDR2HDR by Rempel et al. [7], and *γ* expansion by Masia et al. [4]. The image quality metric presented by Aydin et al. [12] is used to assess the quality of the generated HDR images. The metric generates a summary image with red, green, and blue pixels. Red pixels indicate contrast reversal (the contrast polarity is reversed in the test image with respect to the reference image), green pixels indicate loss of visible contrast (visible contrast in the reference image becomes invisible in the test image), and blue pixels indicate amplification of invisible contrast (not visible in the reference image and visible in the test image). In the experiments, the reference image is the original LDR image; the test image is the generated HDR image.

**Figure 2:**
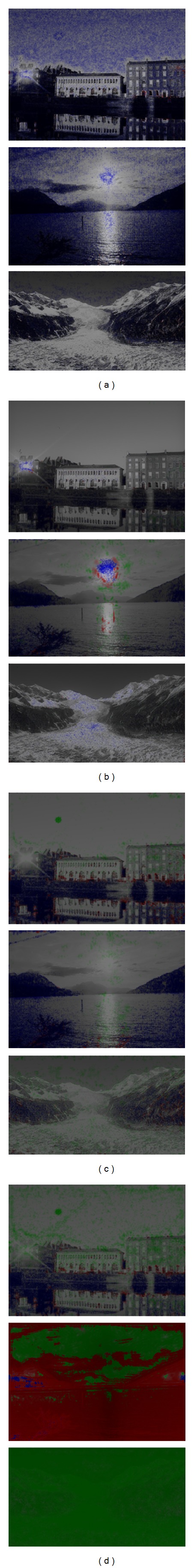
The result images from the metric of Aydin et al. [12]. The original images are building, sun, and snow in Figure 1, (a) proposed, (b) iPG, (c) LDR2HDR, (d) *γ* expansion. Red, green, and blue identify contrast reversal, loss of visible contrast, and amplification of invisible contrast, respectively.

**Figure 3:**
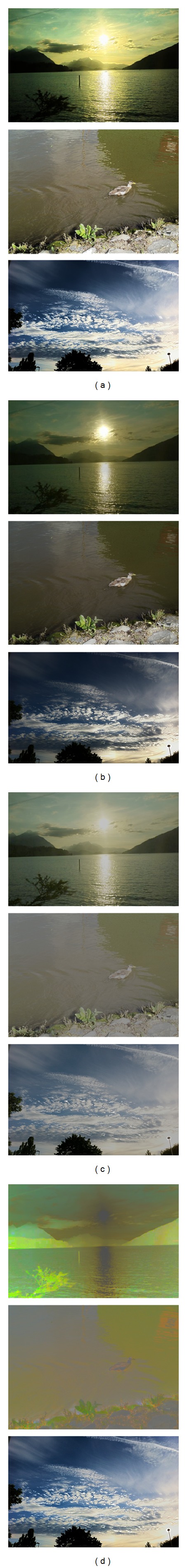
Tone mapped images of four algorithms, with Reinhard et al.'s operator [13]: (a) proposed, (b) iPG, (c) LDR2HDR, and (d) *γ* expansion.

Figure 2 shows some images generated by the image quality metric [12]. By computing the percentage of red, green, and blue pixels (the ratio of the red, green, and blue pixel number to the total number of images) of these metric images, the algorithms also can be compared numerically.  Table 1 displays the percentage values corresponding to images of subset in Figure 1. Because of limitations of the print medium, HDR images cannot be showed here directly. A part of tone mapped images of four algorithms is presented in Figure 3, with Reinhard et al.'s photographic tone mapping operator [13].

Several results of Masia et al.'s algorithm are unpleasant; it is due to the small key values of the LDR images which result in negative *γ* values.  Table 1 indicates that the proposed algorithm has larger blue percentage and smaller red and green percentages, which means that it can disclose more details with little negligible contrast loss and reversal than the other three algorithms; this is visually testified by Figure 2. Figure 3 also shows that the proposed algorithm works well and obtains more pleasing images. In brief, the simulation results prove that, thanks to its physiology and local adaptive properties, the proposed algorithm has good performance for various light conditions or incorrectly exposed images.

## 4. Conclusion

This paper presented a compact ITM algorithm designed for legacy LDR images. It has low computational complexity and can obtain high quality HDR images with a few parameters compared to other recent methods. The imitation of the HVS property and the utilization of adaptive local luminance and the utilization of adaptive local luminance help the algorithm to realize two main goals of an ITM operator: preserving global image details and enhancing local contrasts. The algorithm works well for images with incorrectly exposed areas thanks to its physiological property and enhances more details with little contrast loss and reversal than the methods which largely boost or sophisticatedly deal with the saturated regions. The computational efficiency combined with the high visual quality of the results makes the proposed scheme attractive.

## Figures and Tables

**Table 1:** Red, green, and blue percentages of metric result for subset images: large red percentage means more contrast reversal, large green percentage denotes more contrast loss, and large blue percentage indicates more detail revelation.

Images	Percentage (%)											
	Proposed			iPG			LDR2HDR			*γ* expansion		
	Red	Green	Blue	Red	Green	Blue	Red	Green	Blue	Red	Green	Blue
Sky	1.020	0.072	88.46	6.260	2.140	24.56	7.200	28.71	17.38	3.160	0.380	54.18
Sun	0.370	0.760	81.17	3.040	17.50	31.92	1.620	15.07	43.58	73.15	26.12	0.660
Snow	1.540	0.590	68.09	3.490	9.650	41.44	6.600	55.56	17.15	0	100	0
Sea	1.250	3.350	49.56	2.020	3.160	53.63	2.240	65.05	9.820	1.700	3.040	30.84
Building	1.560	0.190	61.06	3.260	5.250	32.59	8.520	25.90	28.73	6.230	61.70	22.28
Gate	2.680	0.350	76.19	9.100	10.37	65.69	13.75	16.98	40.84	7.820	76.22	10.86
River	2.320	0.760	55.75	4.990	2.200	2.470	10.01	39.93	1.580	24.03	75.92	0

Average	1.534	0.868	68.61	4.594	7.181	36.04	7.134	35.31	22.73	16.59	49.05	16.97

## References

[1] Kim K, Bae J, Kim J (2011). Natural HDR image tone mapping based on retinex. *IEEE Transactions on Consumer Electronics*.

[2] Banterle F, Debattista K, Artusi A (2009). High dynamic range imaging and low dynamic range expansion for generating HDR content. *Computer Graphics Forum*.

[3] Akyüz AO, Fleming R, Riecke BE, Reinhard E, Bülthoff HH (2007). Do HDR displays support LDR content?: a psychophysical evaluation. *ACM Transactions on Graphics*.

[4] Masia B, Agustin S, Fleming RW, Sorkine O, Gutierrez D (2009). Evaluation of reverse tone mapping through varying exposure conditions. *ACM Transactions on Graphics*.

[5] Meylan L, Daly S, Süsstrunk S The reproduction of specular highlights on high dynamic range displays.

[6] Banterle F, Ledda P, Debattista K, Chalmers A Expanding low dynamic range videos for high dynamic range applications.

[7] Rempel AG, Trentacoste M, Seetzen H (2007). Ldr2Hdr: on-the-fly reverse tone mapping of legacy video and photographs. *ACM Transactions on Graphics*.

[8] Guarnieri G, Marsi S, Ramponi G (2011). High dynamic range image display with halo and clipping prevention. *IEEE Transactions on Image Processing*.

[9] Dowling JE (1987). *The Retina: An Approachable Part of the Brain*.

[10] Shapley R, Enroth-Cugell C (1984). Chapter 9: visual adaptation and retinal gain controls. *Progress in Retinal Research*.

[11] Durand F, Dorsey J (2002). Fast bilateral filtering for the display of high-dynamic-range images. *ACM Transactions on Graphics*.

[12] Aydin TO, Mantiuk R, Myszkowski K, Seidel H-P (2008). Dynamic range independent image quality assessment. *ACM Transactions on Graphics*.

[13] Reinhard E, Stark M, Shirley P, Ferwerda J (2002). Photographic tone reproduction for digital images. *ACM Transactions on Graphics*.

